# Rapid anti‐HPA‐1a antibody quantification with a Luminex bead‐based assay: A method evaluation

**DOI:** 10.1111/vox.70180

**Published:** 2026-02-03

**Authors:** Klara Asplund Högelin, Emöke Deschmann, Petter Höglund, Agneta Wikman

**Affiliations:** ^1^ Department of Clinical Immunology and Transfusion Medicine Karolinska University Hospital Stockholm Sweden; ^2^ Department of Laboratory Medicine Karolinska Institutet Stockholm Sweden; ^3^ Department of Women's and Children's Health Karolinska Institutet Stockholm Sweden; ^4^ Department of Neonatal Medicine Karolinska University Hospital Stockholm Sweden; ^5^ Department of Medicine Huddinge Karolinska Institutet Stockholm Sweden

**Keywords:** foetal and neonatal alloimmune thrombocytopaenia, HPA‐1a antibodies, HPA‐1a antibody quantification, human platelet antigen, laboratory diagnostics

## Abstract

**Background and Objectives:**

High concentration of human platelet antigen‐1a (HPA‐1a) antibodies is reported to be associated with severe foetal and neonatal alloimmune thrombocytopaenia (FNAIT). The gold standard for quantification of anti‐HPA‐1a antibodies is the monoclonal antibody immobilization of platelet antigen (MAIPA) assay, which is a laborious method performed in only a few reference laboratories. The aim of this study was to evaluate the performance of the commercially available bead‐based Luminex assay PakLx (Immucor) for quantitative measurement of anti‐HPA‐1a antibodies.

**Materials and Methods:**

We analysed anti‐HPA‐1a antibody levels in plasma samples from 42 HPA‐1a‐negative women who had given birth to a child with thrombocytopaenia. Quantification of antibodies was performed with two different techniques: MAIPA analysed by spectrophotometry with results expressed in international units (IU)/mL, and PakLx analysed in the Luminex assay with results expressed as the mean fluorescence intensity (MFI).

**Results:**

In the comparison of the two methods' ability to stratify a result as either positive or negative, PakLx demonstrated 97.6% agreement with the MAIPA assay, with positive and negative predictive values of 96.7% and 100%, respectively. The correlation of the MFI values from PakLx with IU/mL in MAIPA assay was high, with a correlation coefficient ( *R* ^2^ ) of 0.92. MFI values were converted into semi‐quantitative results: high, intermediate and low levels of anti‐HPA‐1a.

**Conclusion:**

PakLx shows high agreement with the MAIPA assay and allows fast laboratory turnaround time for the determination of anti‐HPA‐1a antibody levels. The result may be of predictive value in clinical assessments.

**Highlights**

- There is a high correlation between the PakLx assay and the monoclonal antibody immobilization of platelet antigen (MAIPA) assay in anti‐human platelet antigen‐1a antibody quantification.
- Quantification with PakLx enables fast laboratory turnaround, with results available within hours.
- The PakLx assay exhibits some lot‐to‐lot variation, but trends can easily be monitored over time.

## INTRODUCTION

Foetal and neonatal alloimmune thrombocytopenia (FNAIT) is an immune‐mediated disorder caused by transplacental passage of maternal‐derived immunoglobulin G (IgG) antibodies directed against paternally inherited foetal platelet antigens. These alloantibodies mediate platelet destruction, leading to foetal thrombocytopaenia, which can manifest as petechiae, bruising or more severe bleeding, including life‐threatening intracranial haemorrhage [1]. In White populations, the majority of FNAIT cases (~80%–90%) are caused by antibodies directed against the human platelet antigen 1a (HPA‐1a) [2, 3], located on the fibrinogen‐binding glycoprotein IIb/IIIa (GPIIb/IIIa) complex, which is essential for platelet aggregation and blood clot formation [4]. The severity of FNAIT is correlated with maternal anti‐HPA‐1a antibody levels: higher antibody concentrations being associated with more profound foetal thrombocytopaenia and an increased risk of serious complications compared to lower concentrations [5, 6, 7]. Thus, quantification of maternal anti‐HPA‐1a antibodies, in terms of antibody levels, can serve as a predictive marker of disease severity and aid in monitoring and guiding prenatal management strategies. The monoclonal antibody immobilization of platelet antigen (MAIPA) assay has for long been the gold standard for anti‐HPA antibody detection and quantification due to its high sensitivity and specificity [8, 9]. However, this method is laborious and time‐consuming, resulting in long laboratory turnaround time. In this project, we aimed to evaluate how quantitative analysis of anti‐HPA‐1a antibodies with MAIPA correlated with MFI values in the bead‐based Luminex assay PakLx (Immucor), which could potentially replace or complement the MAIPA assay to provide faster turnaround times.

## MATERIALS AND METHODS

### Patient cohort and samples

EDTA‐plasma samples were collected from a total of 42 HPA‐1a‐negative women who had given birth to a child with thrombocytopaenia and been referred to the Thrombocyte and Leukocyte‐lab (TRoLL) at the Karolinska University Hospital (Stockholm, Sweden) between 2009 and 2023 for platelet antibody analysis. The plasma samples were stored in −20°C after the initial analysis. Of the 16 samples collected between 2009 and 2014, 7 were re‐analysed by MAIPA at the time of the PakLx assay in 2023. The study was approved by the Swedish ethical review authority (no. 2020‐03821).

### Anti‐HPA‐1a quantification with MAIPA

Quantification of anti‐HPA‐1a antibody levels was performed with an MAIPA assay modified after the procedure described by Kiefel et al. [8], using biotinylated monoclonals and streptavidin‐coated enzyme‐linked immunosorbent assay plates [10]. In short, HPA‐1a homozygous platelets, previously frozen at −80°C, were thawed and incubated with plasma samples alongside an eight‐point standard curve, including a blank, for 45 min at 37°C. The eight‐point standard curve was prepared from the WHO International Anti‐HPA‐1a Standard (NIBSC code: 03/152). It was first diluted to 2500 IU/L in MAIPA buffer (0.5% bovine serum albumin (BSA) in Dulbecco's phosphate‐buffered saline (PBS) with Mg^2+^ and Ca^2+^), followed by 1:3 serial dilution. Plasma samples were diluted to 1:10, 1:50, 1:250 and 1:1250 in MAIPA buffer. The assay range was 1–625 IU/mL for results obtained from at least two sample dilutions. The lower limit of quantification was 0.1 IU/mL for samples diluted to 1:10. Afterwards, the plate was washed twice, followed by incubation with biotinylated anti‐CD41 (clone P2, directed against GPIIb/IIIa, Beckman Coulter) for 45 min at 37°C. The platelets were washed twice, mixed with the lysis buffer (0.5% Triton X‐100 in PBS), incubated for 20 min at room temperature (RT) and then centrifuged at 1000×*g* for 20 min at 10°C. The lysate was transferred to a streptavidin‐coated plate (Immobilizer Streptavidin F96 microplate, Fisher Scientific) and incubated for 60 min at RT on a platform shaker at 300 rpm. After washing, the wells were incubated with HRP‐conjugated goat anti‐human IgG H + L (109‐035‐088, Jackson ImmunoResearch) for 30 min at RT on a platform shaker at 300 rpm. After washing, the substrate was added, followed by the stop solution. The absorbance was measured at 450 nm using a SpectraMax plate reader (Molecular Devices).

### Anti‐HPA quantification with PakLx

The bead‐based multiplex Luminex kit LIFECODES PakLx (ImmuCor) was used for the detection and quantification of anti‐HPA‐1a antibodies, as well as antibodies against other antigens located on GPIIb/IIIa, GPIb/IX, GPIa/IIa, GPIV and HLA Class I, which are included in the assay. The analysis was performed according to the manufacturer's instructions. For each analysis, positive and negative controls provided by the manufacturer were used in addition to the laboratory's in‐house positive anti‐HPA‐1a control. After the reaction, the mean fluorescence intensity (MFI) of each bead was analysed on a Luminex 200 system (Luminex Corporation), according to normal routine. The MATCH IT! antibody software program (Immucor) was used for analysis and interpretation. To assess levels of anti‐HPA‐1a antibodies, the MFI value of the HPA‐1a3a4a bead was used.

### Assessment of lot‐to‐lot variation in PakLx

To assess the lot‐to‐lot variation in PakLx, the WHO international anti‐HPA‐1a standard (100 IU; NIBSC code: 03/152) was diluted in PBS containing 0.2% BSA to form two calibrators (4 and 2 IU/mL). These were used as calibrators each time a new reagent lot was introduced in clinical routine during the years 2022–2025.

### Statistical analysis

Statistical analyses were performed with GraphPad Prism software version 10.3.1. The correlation between the MAIPA (international units [IU]/mL) and the PakLx (MFI) results for anti‐HPA‐1a antibodies was assessed using a five‐parameter logistic curve‐fitting model. To assess lot‐to‐lot variation in PakLx, Spearman's *r* was used. A *p*‐value of ≤0.05 was considered significant.

## RESULTS

### Evaluation of PakLx assay accuracy and concordance with MAIPA

Plasma samples from 42 HPA‐1a negative women were analysed for anti‐HPA‐1a antibodies using the two different methods, namely PakLx and MAIPA. Twelve samples (29%) were negative for anti‐HPA‐1a with PakLx. Of these, seven were below the lower limit of detection (<0.1 IU/mL) and five had a low concentration of anti‐HPA‐1a (<1 IU/mL) in the MAIPA assay. One sample with undetectable levels in the MAIPA assay (<0.1 IU/mL) was positive with PakLx (MFI: 2131). Overall, PakLx demonstrated 97.6% agreement with the MAIPA assay, with positive and negative predictive values of 96.7% and 100%, respectively (Table 1). This agreement was also true when only older samples, collected between 2009 and 2014, were analysed (data not shown).

**TABLE 1:** Detection of anti‐human platelet antigen‐1a antibodies by PakLx and monoclonal antibody immobilization of platelet antigen.

PakLx	MAIPA		
	Positive (>1 IU/mL)	Negative (<1 IU/mL)	Total
Positive	29	1^a^	30
Negative	0	12	12
Total	29	13	42

Abbreviations: IU, international units; MAIPA, monoclonal antibody immobilization of platelet antigen.

^a^ Discrepant result.

### Correlation of anti‐HPA‐1a antibody levels measured by PakLx and MAIPA

**FIGURE 1:**
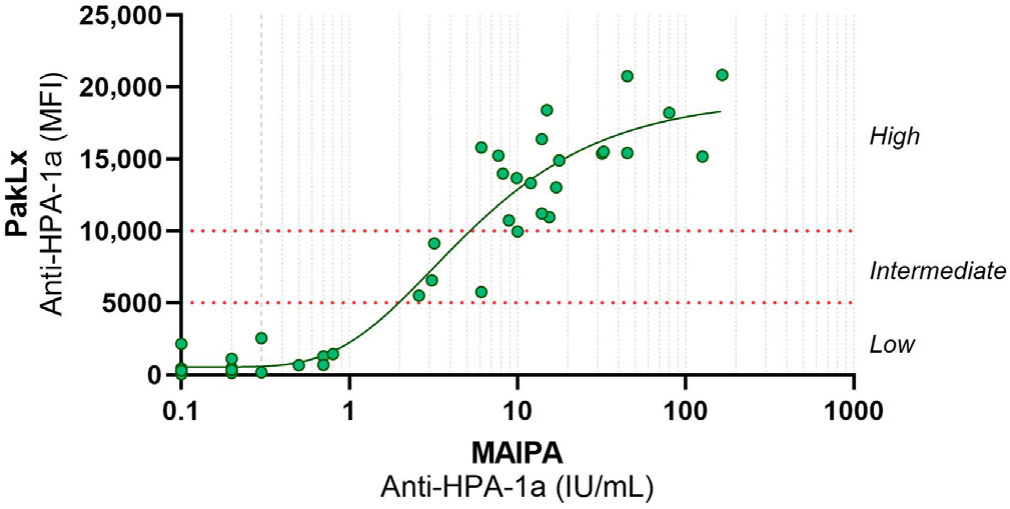
Correlation of anti‐human platelet antigen‐1a (HPA‐1a) antibody levels between the PakLx and the monoclonal antibody immobilization of platelet antigen (MAIPA) assay (*n* = 42). Dotted lines indicate the established cut‐off values that were determined for the PakLx test results to be reported as either low, intermediate or high anti‐HPA‐1a concentration. IU, international units; MFI, mean fluorescence intensity.

When correlating the MFI values from PakLx with the concentrations from the MAIPA assay with a five‐parameter logistic curve‐fitting model, a correlation coefficient (*R* ^2^) of 0.92 was obtained. Based on a previous prospective study showing that maternal anti‐HPA‐1a antibody levels of 3 IU/mL are associated with an increased risk of severe FNAIT [6], and considering that other studies have used higher cut‐off values of 20–30 IU/mL to define increased risk [11, 12], two cut‐off values were defined for the PakLx assay to enable semi‐quantitative reporting in clinical routine diagnostics: a low threshold of 5000 MFI (equivalent to 2 IU/mL) and a high threshold of 10,000 MFI (equivalent to 10 IU/mL), allowing test results to be categorized as low (<2 IU/mL), intermediate (2–10 IU/mL), or high (>10 IU/mL) anti‐HPA‐1a concentrations (Figure 1). The results were reported as low, intermediate or high concentration, without the risk of interpretation.

### Lot‐to‐lot variation in PakLx

**FIGURE 2:**
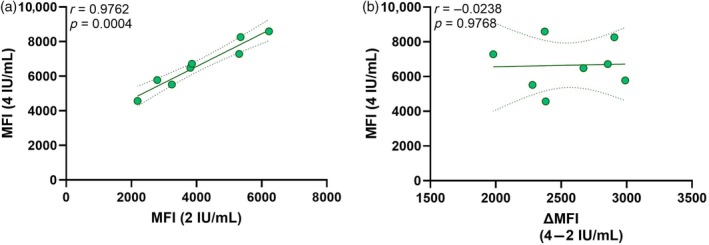
Assessment of lot‐to‐lot variation in PakLx. (a) Correlation between the mean fluorescence intensity (MFI) values corresponding to anti‐human platelet antigen‐1a (HPA‐1a) antibody levels of 4 and 2 IU/mL, measured at each batch change of PakLx (*n* = 8). (b) Correlation between the MFI values at 4 IU/mL and the delta (∆) MFI value, that is, the calculated difference in MFI between the 4 and 2 IU/mL calibrators, at each batch change (*n* = 8). Spearman *r* and *p*‐values are shown. *p*‐values ≤0.05 were considered significant.

One concern when using commercially available kits is lot‐to‐lot variation. Thus, for the PakLx assay, the WHO international anti‐HPA‐1a standard was used as a calibrator at each batch change. The MFI values corresponding to 4 and 2 IU/mL were measured for each new lot (*n* = 8) and displayed a mean (±SD) MFI value of 6653 (±1372) and 4098 (±1403), respectively. The inter‐assay variability, calculated as the coefficient of variation, was 20.6% and 34.2%, respectively. The median (min; max) difference in MFI between the two calibrators was 2671 (1981; 2989) with a standard error of mean of 125.6 and a coefficient of variation of 13.9%. Although there was a strong correlation between the MFI values corresponding to 4 IU/mL and those corresponding to 2 IU/mL (*r* = 0.9762, *p* = 0.0004) (Figure 2a), the MFI values for the 4 IU/mL calibrator did not correlate with the MFI differences between the two calibrators (*p* = 0.98) (Figure 2b). Thus, despite some inter‐assay variability, the intercorrelation and separation between the two calibrators are consistent.

### Routine diagnostics of FNAIT

**FIGURE 3:**
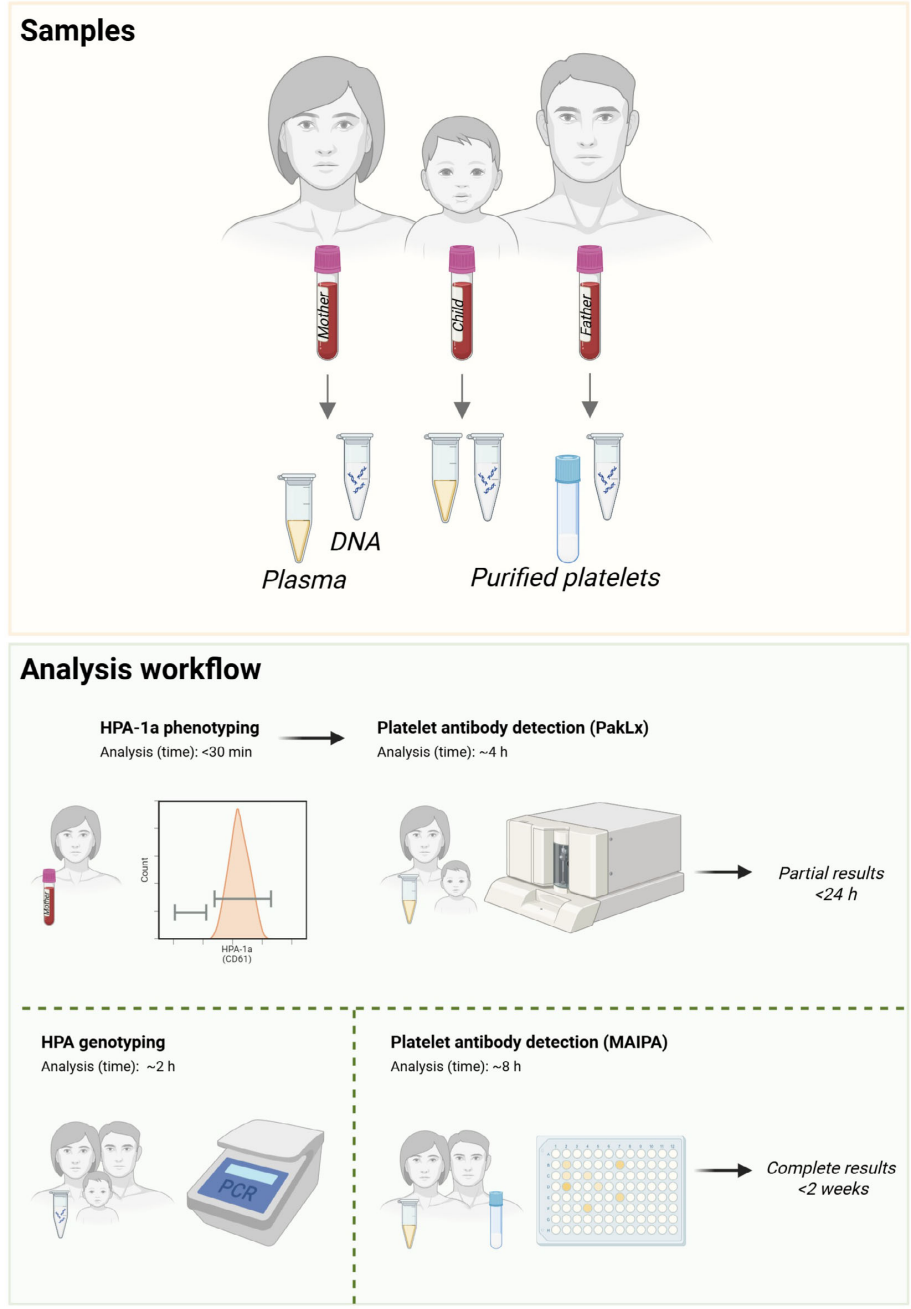
Routine diagnostic workflow for foetal and neonatal alloimmune thrombocytopaenia (FNAIT). One EDTA tube is collected from each family member: mother, child and father. Plasma samples are prepared from both mother and child, while platelets are isolated from the father. DNA is extracted from all family members. Human platelet antigen‐1a (HPA‐1a) phenotyping and PakLx analysis constitute the initial diagnostic tests. This is followed by a confirmatory or more detailed analysis of maternal anti‐HPA antibodies using monoclonal antibody immobilization of platelet antigen (MAIPA). The findings, together with the HPA genotyping results for the entire family, are compiled into the final report. Created with Biorender.com.

At the Clinical Immunology Laboratory at Karolinska University Hospital in Huddinge, Sweden, quantitative results from PakLx are now fully integrated into the routine diagnostic workflow for FNAIT (Figure 3). Together with HPA‐1a phenotyping via flow cytometry, it provides clinicians with a rapid antibody result within 24 h of sample arrival. This report indicates whether the mother is HPA‐1a negative and whether any anti‐HPA antibodies are detected by PakLx, and if anti‐HPA‐1a antibodies are present, the concentration level is reported as low, intermediate or high based on the MFI value. For the final and complete results, HPA genotyping is conducted for the entire family. Furthermore, a confirmatory or extended analysis of maternal anti‐HPA antibodies is performed by MAIPA, including assessment of antibodies against HPA‐15 and antibodies specifically targeting paternal platelets. The final report is issued within 2 weeks after the samples arrive at the laboratory. When anti‐HPA‐1a quantification is requested in antenatal monitoring of HPA‐1a immunized pregnancies, the results are reported as low, intermediate or high concentration, and can consistently be followed during the pregnancy.

## DISCUSSION

In this study, we evaluated the performance of the PakLx assay in determining the level of anti‐HPA‐1a antibodies and assessed its concordance with the MAIPA assay. The observed high agreement between the two assays (97.6%) in the classification of samples as anti‐HPA‐1a positive or negative is in line with previous studies [13, 14, 15, 16]. Only one sample showed a discrepancy, with undetectable levels of anti‐HPA‐1a in the MAIPA assay (<0.1 IU/mL) but the MFI value in PakLx that was defined as positive, although it fell within the ‘low level’ classification with an MFI value below 5000. Several factors may have contributed to this discrepancy. Although both methods target anti‐HPA‐1a antibodies, PakLx employs recombinant antigens coupled to beads and presented in a different conformation, potentially enabling the detection of antibodies not recognized by the native platelet glycoprotein complexes used in MAIPA. In addition, the biotinylated detection antibody used in MAIPA may compete with or bind to the same epitope as the patient's antibody, potentially reducing the signal and leading to a negative result.

Besides the high qualitative agreement in anti‐HPA‐1a detection, the two methods also displayed a strong correlation of quantitative anti‐HPA‐1a antibody levels. The association between high maternal anti‐HPA‐1a antibody levels and the severity of FNAIT supports the relevance of antibody levels as a prognostic indicator. Indeed, several studies have previously reported a correlation between maternal anti‐HPA‐1a antibody levels and platelet counts in the newborn [5, 6]. Additionally, in an early prospective study, Killie et al. identified a maternal anti‐HPA‐1a antibody level of 3 IU/mL as a clinically relevant threshold associated with an increased risk of severe FNAIT [6], and this cut‐off has since been adapted into clinical protocols [17]. More recent studies, however, suggest that substantially higher antibody levels may be more closely associated with the most severe outcomes [11, 12]. In Norway, the management of FNAIT incorporates maternal anti‐HPA‐1a antibody levels as a key parameter for risk stratification. In pregnancies without a history of FNAIT‐associated intracranial haemorrhage, anti‐HPA‐1a antibody levels are monitored serially by MAIPA. If concentrations reach or exceed 3 IU/mL, the pregnancy is classified as higher risk, prompting intensified foetal monitoring and planned delivery by elective caesarean section to minimize the risk of trauma‐related bleeding [17]. However, guidelines regarding mode of delivery differ internationally [18]. In the present study, when screening the 42 HPA‐1a‐negative women, with the two methods, most samples displayed either a high (>10,000) or low (<5000) MFI value, and only a few were within the intermediate area, which may facilitate the assessment of whether the patient is at high or low risk of FNAIT. However, the semi‐quantitative antibody levels measured by PakLx will be prospectively evaluated if they correlate with risk for severe FNAIT in the same way as IU/mL levels measured by MAIPA.

Although this study focuses specifically on the detection of anti‐HPA‐1a antibodies since they are responsible for the vast majority of FNAIT cases, there are more than 30 other anti‐HPA‐specific antibodies that have been reported in FNAIT cases and are of high clinical relevance [19]. Notably, PakLx detects and differentiates between several, but not all known HPAs, namely HPA‐1, HPA‐2, HPA‐3, HPA‐4, HPA‐5 and GPIV. Although this was beyond the scope of the present study, previous reports have demonstrated high concordance between PakLx and other serological methods, such as the MAIPA assay and platelet immunofluorescence test (PIFT), in detecting those anti‐HPA antibodies as well [13, 14, 15, 16, 20]. However, for less frequent or structurally complex antigens, such as HPA‐15, PakLx currently lacks antibody identification beads, and consequently, MAIPA is the preferred method in cases where there is clinical suspicion of other anti‐HPA antibodies not included in the PakLx panel, as it allows for the detection of maternal antibodies against paternal or foetal platelets [21, 22]. However, there are also several methodological considerations specific to the MAIPA assay that must be considered. In the MAIPA assay, platelets from different donors are used as test cells. This introduces variability since HPA‐1a expression levels can differ between individuals, potentially affecting assay sensitivity and reproducibility. Furthermore, there is a potential risk of false‐negative results if the monoclonal antibodies used to detect specific platelet antigens bind to the same or overlapping epitopes as the patient's antibodies. The respective limitations of each method must be carefully considered when choosing the appropriate assay, and the two methods can complement and validate each other, especially in the detection of weak or rare antibodies.

In addition to technical considerations, economic and practical factors are important when selecting a method for routine clinical use. When an economic evaluation comparing the PakLx assay and MAIPA was performed regarding reagent costs, controls as well as hands‐on working time, the overall analysis cost per sample favoured the commercial PakLx assay. For quantitative analysis, PakLx results are generated as part of the screening test without the need for additional samples or reagent costs. In contrast, MAIPA requires repeated analyses with serial dilutions of patient samples. In conclusion, reporting quantitative results from the PakLx assay does not incur additional costs for either the laboratory or the requesting clinicians.

Lastly, although the PakLx assay overcomes several methodological limitations inherent to MAIPA and offers the advantages of reduced labour intensity and faster turnaround times, lot‐to‐lot variation inherent to commercial kits can pose a significant challenge [23]. Here, we assessed the lot variances by measuring two calibrators, corresponding to a concentration of 4 and 2 IU/mL of anti‐HPA‐1a every time a new PakLx lot was used. Indeed, the measured MFI values for the two calibrators varied between the different batches (coefficient of variation = 20.6% and 34.2%, respectively). However, the intercorrelation and separation between the two concentration levels remained consistent. Ideally, this lot‐to‐lot variation should be considered in cases where there is a need to follow the antibody levels over time. However, for the purpose of distinguishing between high and low antibody levels, where pronounced differences in MFI values are observed, the influence of such variation is expected to be limited. That said, this study is not without its limitations. The clinical cut‐off levels for classifying antibody titres as low, intermediate or high could be further refined using a larger patient cohort, alongside the parallel analysis of a comparable standard curve in PakLx as employed in MAIPA. To better investigate and potentially correct for lot‐to‐lot variation, it would have been advantageous to include calibrators with each assay run and adjust accordingly. In addition, even if, generally, the long‐term storage of plasma samples may cause partial degradation of antibodies, there was a strong agreement between the two methods in both classifying samples as positive or negative and in terms of correlation of antibody levels. This suggests that degradation of antibodies was not an issue in this study. Of note, quantification in this study refers solely to anti‐HPA‐1a antibody levels, measured either as IU/mL or MFI, but without any assessment of their function or strength. However, according to previous studies, antibody levels correlate strongly with clinical outcome [5, 6, 7]. Finally, to evaluate how anti‐HPA‐1a antibody levels measured by PakLx can be used to monitor FNAIT, a larger and better characterized cohort, including detailed clinical information on FNAIT severity, neonatal platelet counts and longitudinal samples, would be warranted in future studies.

To conclude, a fast laboratory turnaround time for anti‐HPA‐1a antibody testing and quantification may support the clinical assessment and timely intervention of FNAIT in the antenatal monitoring of immunized pregnancies. We observed a high concordance and strong correlation between the PakLx and the MAIPA assay in the detection and quantification of anti‐HPA‐1a antibodies. Thus, to enhance laboratory response times and ensure quick and accurate anti‐HPA‐1a antibody level assessments, quantitative reporting from the PakLx assay was introduced into clinical routine at the Clinical Immunology Laboratory at Karolinska University Hospital in Huddinge, Sweden, in November 2023.

## CONFLICT OF INTEREST STATEMENT

The authors declare no conflicts of interest.

## Data Availability

The data that support the findings of this study are available from the corresponding author upon reasonable request.
